# Effectiveness of three vestibular rehabilitation exercises for treating acute unilateral peripheral vestibular dysfunction: a multicenter randomized study

**DOI:** 10.3389/fneur.2025.1687181

**Published:** 2026-01-05

**Authors:** Hyun Jin Lee, Dong-Hee Lee, Jae-Hyun Seo, Eun-Jin Son, Eun-Ju Jeon

**Affiliations:** 1 Department of Otorhinolaryngology-Head and Neck Surgery, Incheon St. Mary’s Hospital, College of Medicine, The Catholic University of Korea, Seoul, Republic of Korea; 2 Department of Otorhinolaryngology-Head and Neck Surgery, Uijeongbu St. Mary’s Hospital, College of Medicine, The Catholic University of Korea, Seoul, Republic of Korea; 3 Department of Otolaryngology-Head and Neck Surgery, Seoul St. Mary’s Hospital, College of Medicine, The Catholic University of Korea, Seoul, Republic of Korea; 4 Department of Otorhinolaryngology, Yongin Severance Hospital, Yonsei University College of Medicine, Yongin-si, Republic of Korea

**Keywords:** vestibular rehabilitation, acute unilateral vestibulopathy, balance function, dizziness, vestibulo-ocular reflex

## Abstract

**Background:**

Vestibular rehabilitation therapy (VRT) is an established intervention for patients with acute unilateral peripheral vestibular dysfunction (aUPVD). However, the relative effectiveness of different VRT strategies remains uncertain. This multicenter randomized pilot trial aimed to compare the effects of customized vestibular rehabilitation therapy (CVRT), generic Cawthorne–Cooksey exercises (GVRT), and vestibulo-ocular reflex (VOR) adaptation exercises on vestibular symptom recovery and functional improvement.

**Methods:**

A total of 34 patients with aUPVD were enrolled across three centers, of whom 23 completed the study and were included in the final analysis (CVRT, *n* = 11; GVRT, *n* = 6; VOR, *n* = 6). All participants underwent an 8-week rehabilitation program, performing prescribed exercises three times daily. Outcomes included the Modified Clinical Test of Sensory Interaction on Balance (mCTSIB), Functional Reach Test (FRT), Visual Analog Scale (VAS), Dizziness Handicap Inventory (DHI), Disability Scale (DS), and Activities-specific Balance Confidence (ABC) scale. Nonparametric tests with post-hoc effect size estimation were applied.

**Results:**

Significant within-group improvements were observed in mCTSIB, VAS, DS, DHI, and ABC scores in the CVRT group (*p* < 0.05). The GVRT group showed significant improvements in mCTSIB (foam EC), VAS, DS, DHI, and ABC (*p* < 0.05), whereas the VOR adaptation group showed limited improvement in mCTSIB (foam EO) and DS. Although all three intervention groups demonstrated significant improvements after rehabilitation, no statistically significant differences were found among groups. The CVRT group demonstrated the largest numerical gains in VAS and ABC scores, but these differences did not reach statistical significance.

**Conclusion:**

All three vestibular physical therapy approaches effectively improved symptoms and functional balance in patients with aUPVD. While between-group differences were not statistically significant, the customized rehabilitation group showed the most consistent pattern of improvement. These findings suggest potential clinical advantages of individualized therapy, warranting confirmation in larger studies.

## Introduction

The core theoretical foundation of vestibular rehabilitation therapy (VRT) is central nervous system (CNS) plasticity, which refers to the ability to compensate for peripheral or CNS impairments through sensory and motor neural reorganization (1). Following acute unilateral vestibular loss, CNS plasticity facilitates vestibular compensation via three mechanisms (2): adaptation, which adjusts abnormal tonic afferent signals from peripheral receptors; habituation, which modifies memorized responses to stimuli through experience; and substitution, which utilizes alternative balance strategies, such as saccadic eye movements or visual cues. Static and dynamic vestibular compensation can be achieved (3, 4). Vestibular compensation is a dynamic, multisensory process that restores balance and gaze stability after unilateral vestibular loss. Vestibular compensation involves several interacting mechanisms: (1) restoration of neural symmetry within the vestibular nuclei via spontaneous and adaptive plasticity; (2) sensory substitution, in which visual and somatosensory cues partially replace impaired vestibular input; and (3) habituation and readaptation, achieved through repeated movement exposure and motor learning (5). Vestibular rehabilitation therapy (VRT) primarily enhances these adaptive processes by promoting sensory reweighting, gaze stabilization, and balance control. Vestibular compensation involves neuroplastic reorganization within the vestibular nuclei and cerebellum, contributing to the gradual restoration of postural stability and sensory integration (6).

VRT is typically implemented by training patients on exercise techniques during outpatient visits, followed by repetitive practice at home. VRT can be broadly categorized into generic and customized approaches. Generic VRT applies standardized exercise programs uniformly across patients, irrespective of their individual status. Notable examples include the Cawthorne-Cooksey exercise (CCE), developed in the 1940s, and which has been widely used. CCE is a standardized vestibular rehabilitation program designed to improve gaze stability, postural control, and gait performance through a series of structured and progressive movements. It involves sequentially coordinated exercises of the eyes, head, and body performed in a specific pattern. Originally developed for group-based settings, CCE has been widely used as a generic exercise program in vestibular rehabilitation (7–10). Early initiation of vestibular rehabilitation has been suggested to promote neural plasticity and accelerate recovery after unilateral vestibular loss, provided that exercises are introduced gradually and safely. Previous studies have reported that early mobilization and gaze-stabilization training can enhance compensation after vestibular neuritis (41, 44). However, the optimal timing remains debated, and very early initiation should be applied cautiously depending on symptom tolerance and clinical stability.

The customized VRT is based on the specific symptoms, underlying conditions, and degree of vestibular dysfunction of each patient. It typically includes a combination of therapeutic components, such as eye-head coordination exercises targeting the vestibule-ocular reflex (VOR) through an adaptation mechanism to enhance gaze stability. Balance and gait training are tailored to enhance postural control, reduce fall risk, and improve dynamic stability in daily life. Exercises are selected and modified based on the functional limitations, tolerance, and rehabilitation goals of the patient, allowing for a more targeted and efficient recovery process. These rehabilitation paradigms differ in their therapeutic focus: the Cawthorne–Cooksey exercises emphasize general conditioning and habituation, VOR adaptation focuses on oculomotor recalibration, and customized vestibular rehabilitation targets individualized sensory and postural deficits.

An evidence-based 2016 guideline states that clinicians may employ “specific exercise techniques to target identified impairments or functional limitations” for each patient (11). The updated version for 2022 further recommended that clinics and organizations establish consistent examination and treatment protocols that are customized for the specific vestibular signs and symptoms of the individual (12). Reflecting this growing emphasis on personalized care, the customized VRT approach has gained increasing recognition in recent years because of its clinical effectiveness and patient-centered nature (10, 13, 14).

Several high-quality clinical studies have demonstrated the effectiveness of customized VRT for various vestibular disorders (11, 15, 16), but direct comparisons of customized and generic or other VRT approaches are relatively limited. Further, the results of existing comparative studies are inconsistent; some have reported superior outcomes with customized interventions (17–19), while others have found no significant difference between the approaches (20, 21).

Acute unilateral peripheral vestibular dysfunction (aUPVD), often caused by vestibular neuritis, presents with sudden prolonged vertigo, spontaneous nystagmus, nausea, and postural imbalance without hearing loss. Vertigo and nystagmus usually last for 2–3 days, whereas balance disturbances may persist for 1–2 weeks. Most patients regain the ability to perform basic daily activities within 1–2 weeks (22, 23). However, some patients experience residual dizziness or unsteadiness, especially during movement or in visually complex environments. It has been well-established through numerous studies that vestibular rehabilitation in patients with aUPVD facilitates central compensation, promoting faster recovery from dizziness and an earlier return to daily activities (2, 8). However, relatively few high-quality studies have compared the effectiveness of different vestibular rehabilitation approaches such as customized, generic, or other types of VRT in patients with aUPVD. Most studies on VRT for aUPVD published to date have primarily emphasized the overall benefits of vestibular rehabilitation (24, 25), while few have compared the effects of customized and generic rehabilitation programs (12, 20). Given that each of these rehabilitation strategies is based on distinct principles—adaptation, habituation, and substitution—a systematic evaluation of their relative efficacy is necessary (11). In this study, vestibular rehabilitation refers specifically to exercise-based vestibular physical therapy interventions rather than multidisciplinary rehabilitation approaches such as counseling or pharmacologic management. Understanding whether a specific rehabilitation protocol yields superior outcomes for vestibular function recovery can guide clinical decision-making and improve standardized treatment protocols for acute vestibular dysfunction.

This study aimed to compare the effects of three vestibular rehabilitation exercise approaches, customized VRT, generic VRT, and isolated VOR adaptation exercises, on vestibular function recovery and symptom improvement in patients diagnosed with aUPVD. Through a prospective randomized study, we sought to provide clinical evidence on the most effective VRT strategy for treating acute vestibular dysfunction.

## Participants and methods

### Patients

This prospective, randomized, single-blind, controlled clinical study was conducted at three academic referral hospitals between April 2013 and April 2015. The study adhered to the CONSORT 2010 statement for reporting randomized controlled trials and was conducted according to the Declaration of Helsinki and all its revisions. This study was approved by the Institutional Review Board (IRB approval number: XC12OIMI0147O). All patients provided written informed consent before participation. The study was conducted under full ethical approval before the implementation of mandatory national trial registration for investigator-initiated rehabilitation studies.

Patients with aUPVD seeking care at academic referral hospitals were enrolled in this study. This study included adults aged 18 years who were diagnosed with acute idiopathic vestibular dysfunction. To be eligible, participants were required to have severe rotational vertigo lasting more than 24 h, no accompanying auditory symptoms or signs, canal paresis confirmed through a caloric test, and no other neurological symptoms or signs. The term “acute” was defined as the period within 7 days from the onset of vertiginous symptoms to enrollment, based on the clinical consensus used across the participating hospitals. This corresponds to the early phase of vestibular neuritis when spontaneous nystagmus and postural instability are typically present.

Objective vestibular assessments such as the video head impulse test (vHIT), vestibular-evoked myogenic potentials (VEMPs), or computerized dynamic posturography were not routinely available across all participating centers at the time of this study and therefore could not be implemented uniformly. To maintain methodological consistency, the diagnosis of acute unilateral peripheral vestibular dysfunction (aUPVD) was based on characteristic clinical features, including acute-onset prolonged vertigo, spontaneous horizontal–torsional nystagmus, and postural imbalance without accompanying hearing loss, as assessed by experienced otologists at each institution. This clinical diagnosis was further supported by caloric test results showing unilateral canal paresis greater than 25%, calculated using Jongkees’ formula, which was applied as the diagnostic threshold for vestibular hypofunction.

Participants were excluded if they were under 18 years of age, had suspected or confirmed central vestibular disorders, or had experienced vertigo caused by head trauma (e.g., traffic accidents or falls). Additional exclusion criteria included the presence of coexisting otological conditions (such as otitis media or otosclerosis), recurrent vestibular disorders (such as benign paroxysmal positional vertigo or Ménière’s disease), musculoskeletal conditions that prevented participation in VRT, or refusal to participate in the study.

### Experiment protocol

**Figure 1:**
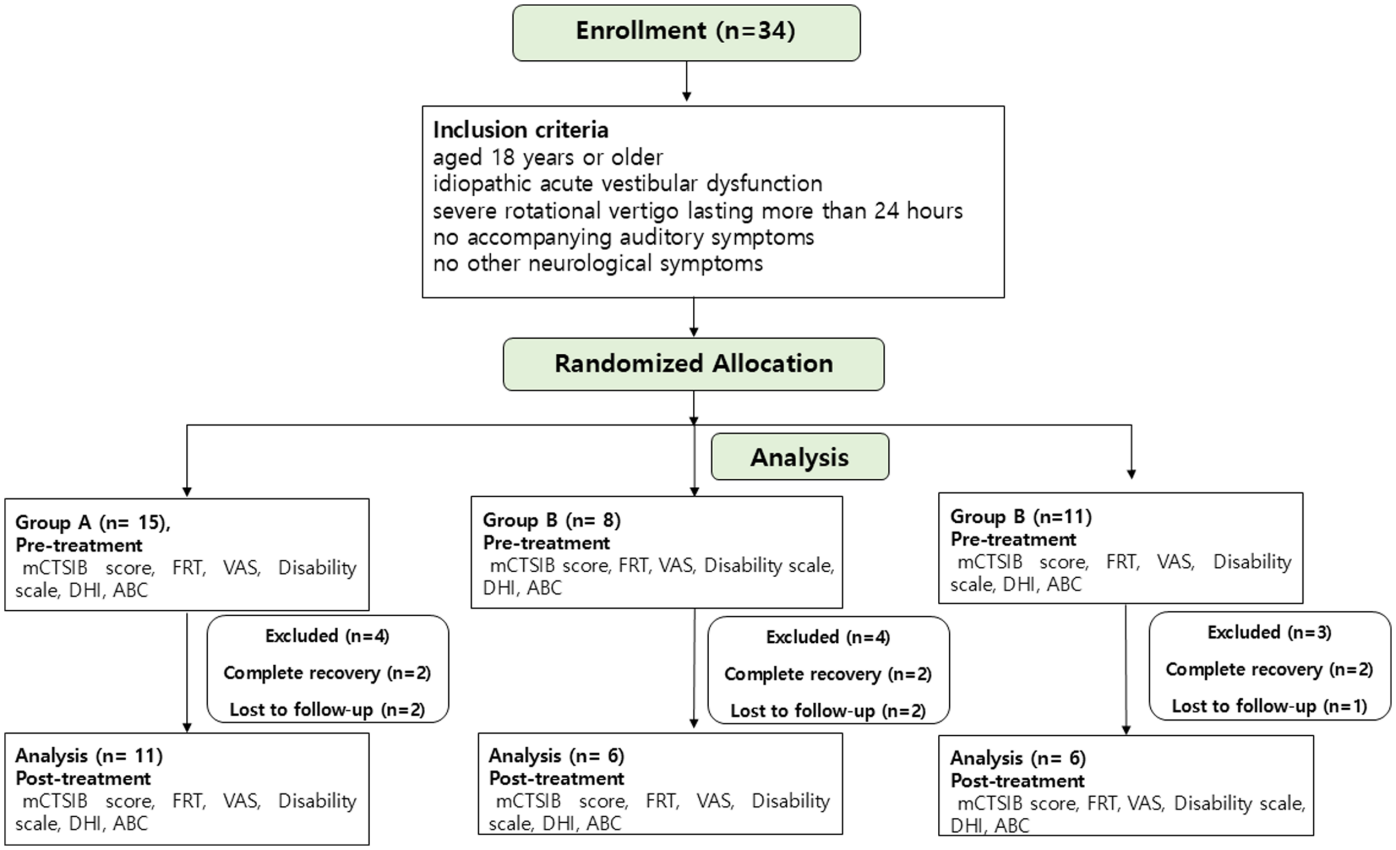
CONSORT flow diagram of patient enrollment and follow-up. A total of 34 patients were enrolled and randomized into three rehabilitation groups (CVRT = 15, GVRT = 8, VORX = 11). Eleven participants did not complete the study (six due to complete symptom resolution and five lost to follow-up), leaving 23 patients who completed the 8-week intervention and were included in the final analysis. Pre- and post-treatment assessments included the modified Clinical Test of Sensory Interaction on Balance (mCTSIB), Functional Reach Test (FRT), Visual Analog Scale (VAS), Disability Scale (DS), Dizziness Handicap Inventory (DHI), and Activities-Specific Balance Confidence (ABC) scale.

Participants were grouped into three based on the type of vestibular rehabilitation exercise: customized VRT (CVRT), generic VRT (GVRT; CCE), and VOR adaptation exercise (VORX). They were allocated to one of the three groups using a block randomization method to ensure comparability and eliminate investigator bias. Randomization was performed by an independent statistician using computer-generated random numbers. The allocation was concealed in sealed envelopes distributed to each participating institution. A single-blind design was implemented: outcome assessors and data analysts were blinded to group allocation, whereas participants were aware of their assigned rehabilitation program but were not informed of the study hypotheses or other group condition (Figure 1).

All groups were educated and trained on the prescribed exercise under weekly supervision during clinic visits. After supervised VRT at the clinic, they were instructed to practice the same exercise at home three to five times daily, with a minimum of 10 min at each time to reach a total duration of more than 30 min per day. Adherence to home exercises was encouraged through verbal reinforcement during clinic visits and patient self-reporting logs; however, objective adherence tracking was not performed, which is acknowledged as a limitation of this study.

Individualized VRT was prescribed to the patients in the CVRT group; it included VOR adaptation exercises and posture and gait training tailored to their specific symptoms and dysfunction. The GVRT group followed a standardized Cawthorne-Cooksey protocol, and they performed exercises three times daily. The VORX group practiced “VORX1” and “VORX2” gaze stabilization exercises three times daily to improve VOR functionality.

Vestibular rehabilitation sessions were conducted by audiology technicians under the supervision of otolaryngologists experienced in vestibular rehabilitation. Each center had at least one designated technician who instructed and monitored the exercises under medical oversight. In Korea, audiology technicians are licensed clinical laboratory technologists trained in audiology and vestibular sciences accredited by the Korean Audiological Society.

### Treatment and follow-up protocol

Vestibular rehabilitation was initiated after the resolution of severe vertigo, which was typically within 1 week of diagnosis, and participants performed exercises three times daily for 8 weeks. During the first 4 weeks, participants attended weekly clinic visits to monitor adherence and progress, followed by biweekly visits during the subsequent 4 weeks for follow-up evaluations. All participants were encouraged to maintain consistency and intensity of home training, as the effectiveness of vestibular physical therapy is known to be dose-dependent. The use of vestibular suppressant medications was minimized; however, dimenhydrinate was allowed on an as-needed basis for severe dizziness, with all instances of use being carefully recorded.

At the end of the 8-week rehabilitation program, the participants underwent comprehensive evaluations to assess the recovery of vestibular function and improvement in symptoms. These evaluations included diagnostic and survey-based assessments. The primary outcome was the change in Dizziness Handicap Inventory (DHI) total score from baseline to week 8, as this measure most directly reflects the impact of dizziness on daily life. Secondary outcomes included changes in the Activity-Specific Balance Confidence (ABC) Scale, Disability Scale (DS), Visual Analog Scale (VAS), Functional Reach Test (FRT), and modified Clinical Test of Sensory Integration and Balance (mCTSIB).

### Assessment

#### Questionnaires

##### Visual analog scale

The VAS was used to quantify the severity of dizziness experienced by patients. Subjective dizziness was rated on a scale of 0–10, where 0 represented the absence of symptoms, and 10 represented the highest possible intensity of symptoms reported by the patient (26).

##### Disability scale

The Disability Scale assesses the overall level of functional limitation due to dizziness in daily life. Patients rated their degree of disability on a 6-point scale ranging from 0 (no symptoms) to 5 (complete disability). Higher scores indicated greater impairment of daily activities. This scale is simple to administer and has been used to quantify functional recovery in patients undergoing vestibular rehabilitation. A reduction of at least one point is generally considered clinically meaningful (27).

##### Korean dizziness handicap inventory

The DHI was designed to assess the challenges due to dizziness that individuals may face in their daily lives. It consists of 25 questions categorized under emotional, functional, and physical domains. Each item was scored as 0, 2, or 4 based on the presence and frequency of symptoms. The total DHI score ranges from 0 to 100, with higher scores indicating more severe symptoms (28). For patients in our study, the DHI questionnaires translated into Korean and validated by the Korean Balance Society was used (29). The minimally clinically important difference for the DHI was set at 18 points (28).

##### Korean activity-specific balance confidence scale

The ABC scale measures the confidence of the patients in performing 16 common body movements daily. Each movement was rated as a percentage, reflecting the perceived confidence of the patient in executing the movement. The average of these percentage scores was calculated as the overall ABC scale score. The validated Korean version of the ABC scale developed by the Korean Balance Society was used in this study (29). The minimally clinically important difference for the ABC scale was 18.1% (30).

#### Physical examination

##### Functional reach test

For the FRT, patients were instructed to stand upright and extend their arms forward while making fists with their palms facing downward. The horizontal distance between the body and third metacarpal was measured. Next, patients were asked to bend their upper body forward as much as possible while keeping their arms parallel to the ground, and the distance between the body and third metacarpal was measured again. The test was performed three times, and the average difference between the two distances was calculated to determine the results.

##### Modified clinical test of sensory integration and balance

The mCTSIB was performed on the floor and on a medium-density compliant foam with the feet together. The participants were instructed to maintain an upright position for up to 30 s during the test. The mCTSIB included four conditions: firm surface with eyes open (firm EO), firm surface with eyes closed (firm EC), foam surface with eyes open (foam EO), and foam surface with eyes closed (foam EC). Each condition was repeated three times, and the average score was used for analysis. Postural stability was assessed based on clinical observation using a 4-point sway grading scale: 1 = minimal sway, 2 = mild sway, 3 = moderate sway (near fall), and 4 = fall or loss of balance.

#### Intervention

Three exercise programs were selected to represent different rehabilitation paradigms—customized, generic, and VOR-based—allowing comparison between tailored, standardized, and focused training approaches.

##### Customized VRT (CVRT group)

The customized vestibular rehabilitation program consisted of a combination of exercises including gaze stabilization, postural stability, and gait training. Specific exercises were prescribed based on the patient’s condition and individual impairments. Each exercise was conducted under standardized conditions and supervised during initial instructions. The patients were then instructed to continue the exercise independently at home.

During weekly follow-up sessions, the rehabilitation contents were reassessed and adjusted according to each patient’s symptom progression and balance performance, ensuring a dynamically tailored and symptom-oriented approach. This adaptive process differentiated the CVRT group from the GVRT and VORX groups, which followed fixed standardized protocols.

(1) Gaze stabilization exercise

- X1 viewing exercise–long distance

Participants were seated and instructed to fix their gaze on a visual target (a printed symbol on paper) affixed to a wall at eye level at a distance of approximately 1 meter. While maintaining a visual fixation on the target, the participants performed horizontal head movements (side-to-side rotations) for 1 min. After a 1-min rest, vertical (up-and-down) and diagonal (left-down to right-up and right-down to left-up) head movements were performed, each for 1 min, with a 1-min rest between directions.

- X1 viewing exercise–short distance

The X1 short-distance viewing exercise followed a similar protocol but was performed using hand-held paper with printed symbols at arm’s length (approximately 30–40 cm from the eyes). While seated, participants were asked to fixate on the symbol while performing the same series of head movements (horizontal, vertical, and diagonal) with equivalent duration and rest intervals.

- X2 Viewing Exercise

The X2 viewing exercise was performed in the sitting position. Participants held a piece of paper with a printed symbol at arm’s length and were instructed to simultaneously move their head and target in opposite directions. For the horizontal (head right while paper left, and vice versa), vertical (head up while paper down, and vice versa), and diagonal (e.g., head right up while paper left down) directions, the participants performed the movement for 1 min, followed by a 1-min rest.

The X1 and X2 viewing exercises were progressively modified by adjusting for variables such as exercise duration, head movement speed, body position (seated vs. standing), and background complexity of the visual target (plain vs. busy backgrounds). A simple combination of parameters was initially selected based on patient tolerance and functional status. As the patients became accustomed to each step, the next level of difficulty was introduced in a graded manner. This progressive approach aims to optimize VOR adaptation and enhance gaze stability by minimizing retinal slip during head movement.

(2) Postural stability exercise

The participants were asked to stand with their feet together, cross their arms over their chest with their hands resting on the opposite shoulders, and maintain an upright posture while looking straight ahead. They were instructed to hold this position for 1 min with their eyes open, followed by 1 min with their eyes closed. The tests were performed on a stable and firm support surface to ensure safety. If the participants were able to maintain their position for more than 1 min without significant postural sway, the difficulty level progressively increased. Progression strategies included extending the standing duration, adding head movements in horizontal, vertical, or diagonal directions, narrowing the base of support by adopting a tandem or heel-to-toe stance, and changing the surface condition from a firm floor to a compliant surface such as a carpet or foam mat. These modifications were applied in a stepwise manner to systematically challenge postural control and promote multisensory integration during the balance tasks.

(3) Gait exercise and environmental adjustments.

To improve gait stability, the participants were prescribed walking exercises twice daily for 30 min. For cases where dizziness was severe or there was a high risk of falling, patients were instructed to begin walking indoors in a safe home environment with the assistance of a caregiver. The walking exercises were initially performed in well-lit, quiet areas with firm, level surfaces at a slow pace. Gait training was progressively intensified by increasing the walking speed and incorporating more challenging conditions such as dim lighting, crowded environments, uneven or soft surfaces, inclined paths, and dual-task performance. Additional tasks were introduced to promote sensory integration, including turning the head or counting backward while walking. Patients were also educated on strategies to reduce fall risk by evaluating and modifying their living environment, including appropriate footwear, use of assistive devices, and safe home adjustments.

##### Cawthorne-Cooksey exercise (GVRT group)

The CCE is typically performed in four sequential positions: lying down, sitting, standing, and walking (10). In the supine position, exercises begin with slow to progressively faster eye movements, including vertical and horizontal gaze shifts and tracking a moving finger positioned approximately 90 cm from the face and moving laterally approximately 30 cm. This is followed by head movements, including flexion, extension, and rotation, performed slowly at first with the eyes open, and then gradually progressing to faster movements with the eyes closed. In the seated position, eye and head movements were repeated in the supine position. Additional exercises include shoulder shrugs and rotations and forward bending to pick up objects from the floor, which helps improve upper-body coordination and dynamic balance. In the standing position, all previously described supine and seated exercises were repeated. The participants also performed sit-to-stand transitions; they had their eyes opened initially and then had them closed to challenge their postural stability. Further tasks included tossing a ball from one hand to the other above eye level and passing a ball below knee level from one hand to the other to enhance motor coordination and dynamic balance. Finally, walking or movement-based exercises were introduced. These involved walking across a room, initially with eyes open and then with eyes closed. They ascended and descended ramps or inclined surfaces under both visual conditions. They also climbed the stairs, first with eyes open and then with eyes closed. The program concluded with functional play-based tasks such as bending, aiming, and throwing in games such as ring toss, bowling, or basketball, which integrate vestibular challenges with real-world motor skills.

##### VOR adaptation exercise (VORX group)

The participants in the VORX group performed only the X1 and X2 viewing exercises using the CVRT protocol. Exercise parameters, including duration, frequency, body position, and methods for progressing difficulty, were the same as those used for the CVRT group. However, the participants in the VORX group did not perform any posture or gait training exercises, unlike those in the CVRT group.

#### Statistical analysis

Statistical analyses were performed to evaluate both inter- and intra-group differences in the vestibular rehabilitation outcomes. Intergroup comparisons were conducted using the Kruskal-Wallis test to assess the differences among the three rehabilitation groups. The inter-group differences between the pre- and post-exercise measurements were analyzed using the Wilcoxon signed-rank test. Because the total sample size was small and the outcome variables were not normally distributed, parametric multifactorial analyses such as two-way ANOVA were not appropriate. Accordingly, nonparametric tests (Wilcoxon signed-rank and Kruskal–Wallis) were adopted to evaluate within- and between-group changes. Effect sizes (η^2^) and post-hoc power calculations were added for major outcome measures to complement the interpretation of non-parametric results in this small-sample study. Based on the observed variability in ΔDHI (Dizziness Handicap Inventory) scores across the three groups, a post-hoc power analysis was conducted using G*Power (version 3.1). The calculated effect size (Cohen’s *f* = 0.30) yielded an achieved power of approximately 0.63 at a two-sided α = 0.05, consistent with the exploratory design of this pilot study. All statistical analyses were conducted using SPSS version 18.0 (SPSS Inc., Chicago, IL, United States). *p*-values less than 0.05 denoted statistical significance.

## Results

A total of 34 patients with acute unilateral peripheral vestibular dysfunction (aUPVD) were initially enrolled and randomly allocated into three intervention groups: Customized Vestibular Rehabilitation (CVRT, *n* = 15), Generic Cawthorne–Cooksey Exercises (GVRT, *n* = 8), and VOR Adaptation Exercises (VORX, *n* = 11). During the 8-week rehabilitation period, 11 participants did not complete the program — six discontinued early due to complete resolution of dizziness and imbalance, and five were lost to follow-up. Consequently, data from 23 patients (CVRT = 11, GVRT = 6, VORX = 6) were included in the final analysis (Figure 1). The distributions of sex and the affected ear (right vs. left) were comparable for the groups (Table 1). The absolute mean (± SD) canal paresis percentages for each group were as follows: CVRT, 13.2 ± 7.1%; GVRT, 13.7 ± 6.4%; VORX, 14.1 ± 7.5% (see Table 1). There were no statistically significant differences in mean CP% between groups. Although the overall sample size was limited, baseline characteristics were well balanced among the groups, supporting the comparability of study arms.

**Table 1:** Demographic data of the participants.

Variables	Group CVRT (*n* = 11)	Group GVRT (*n* = 6)	Group VORX (*n* = 6)
AUPVD	11	6	6
Age (SD)	47 (14)	52 (16)	57 (10)
Gender (M: F)	6: 5	3: 3	1: 5
Affected ear (Right: Left)	5: 6	2: 4	4: 2
Canal paresis (%)	13.2 ± 7.1%	13.7 ± 6.4%	14.1 ± 7.5%

Group CVRT, Customized exercise; Group GVRT, Cawthorn-Cooksey exercise; Group VORX, VOR adaptation exercise; AUPVD, Acute unilateral peripheral vestibular dysfunction.

Pretreatment assessments revealed no significant differences among the groups in terms of the mCTSIB scores, FRT, VAS, DS, DHI, and ABC scale (*p* > 0.05). The DHI-total score was higher for the GVRT group (72.67 ± 17.83) than for the CVRT (55.45 ± 36.02) and VORX (41.00 ± 35.97) groups; however, the difference was not statistically significant.

Following the 8-week intervention, significant improvements were observed in several outcome measures for the CVRT group. This group demonstrated reductions in postural instability under the mCTSIB conditions with eyes closed on a firm surface or foam surfaces (firm EC, foam EO, and foam EC; *p* = 0.041, *p* = 0.026, and *p* = 0.002, respectively). Reductions in dizziness severity (VAS, *p* = 0.001), self-perceived disability (DS, *p* = 0.008), and total DHI score and its subdomains (all *p* < 0.01) were also observed. Balance confidence, as assessed using the ABC scale, also improved significantly (*p* = 0.001). The mean reduction in DHI-total for the CVRT group exceeded the minimally clinically important difference (MCID = 18 points), suggesting a clinically meaningful improvement in perceived handicap. For the GVRT group, significant improvements were also observed under the foam EC condition of the mCTSIB (*p* = 0.004), along with reductions in the VAS (*p* = 0.013), DS (*p* = 0.005), and DHI total and subdomain scores (all *p* < 0.01). The ABC scores increased significantly after the treatment (*p* = 0.002). The VORX group showed significant changes in the mCTSIB foam EO (*p* = 0.048) and DS (*p* = 0.049), with a trend toward improvement in VAS (*p* = 0.051); however, changes in DHI and ABC did not reach statistical significance (Table 2). Across all groups, the direction of change was consistent with clinical recovery, although the magnitude of improvement varied.

**Table 2:** Comparison of vestibular and balance function before and after treatment.

Measure	Group CVRT (*n* = 11)		*p*-value	Group GVRT (*n* = 6)		*p*-value	Group VORX (*n* = 6)		*p*-value
	Baseline	Post treatment		Baseline	Post treatment		Baseline	Post treatment	
mCTSIB firm EO	1.55 ± 1.04	1.09 ± 0.30	0.138	1	1	1.000	1	1	1.000
mCTSIB firm EC	2.09 ± 1.30	1.09 ± 0.30*	0.041	1.67 ± 0.52	1.67 ± 0.52	1.000	1.50 ± 0.55	1.33 ± 0.52	0.564
mCTSIB foam EO	2.27 ± 1.27	1.27 ± 0.65*	0.026	2.00 ± 0.63	1.50 ± 0.55	0.203	2.17 ± 0.408	1.17 ± 0.41*	0.048
mCTSIB foam EC	3.09 ± 1.04	1.64 ± 1.03*	0.002	3.00 ± 0.63	2.17 ± 0.75*	0.004	3.33 ± 0.82	2.00 ± 1.10	0.068
FRT	26.18 ± 11.13	27.52 ± 9.30	0.655	31.83 ± 11.05	27.17 ± 10.11	0.238	30.50 ± 5.54	33.55 ± 7.04	0.273
VAS	7.45 ± 2.84	0.73 ± 1.27*	0.001	7.83 ± 2.64	1.50 ± 1.64*	0.013	6.33 ± 3.50	2.50 ± 3.15	0.051
Disability scale	2.36 ± 1.36	0.64 ± 0.92*	0.008	3.33 ± 1.37	0.67 ± 0.82*	0.005	2.17 ± 1.72	0.33 ± 0.52*	0.049
DHI-total	55.45 ± 36.02	9.27 ± 15.71*	0.003	72.67 ± 17.83	9.67 ± 10.46*	0.002	41.00 ± 35.97	10.33 ± 12.74	0.116
DHI-functional	20.55 ± 14.31	3.09 ± 10.25*	0.007	32.33 ± 7.09	3.67 ± 4.63*	0.001	17.33 ± 16.57	2.67 ± 5.61	0.116
DHI-emotional	16.91 ± 14.12	2.55 ± 8.44*	0.008	21.33 ± 9.61	2.33 ± 2.34*	0.010	13.33 ± 13.25	3.67 ± 4.46	0.144
DHI-physical	18.00 ± 10.04	3.64 ± 7.69*	0.002	19.00 ± 3.52	3.67 ± 3.88*	0.002	10.33 ± 7.53	4.00 ± 3.80	0.085
ABC	36.73 ± 33.73	93.36 ± 11.72*	0.001	36.68 ± 18.65	84.83 ± 20.06*	0.002	69.90 ± 21.84	85.52 ± 19.34	0.116

Group CVRT, Customized exercise; Group GVRT, Cawthorn-Cooksey exercise; Group VORX, VOR adaptation.

Within-group differences of pre- and post- exercise: Wilcoxon signed rank test, *p < 0.05: significant.

Inter-group comparisons of change scores (*Δ*scores) did not reveal statistically significant differences in any measure. The largest numerical improvements in VAS and ABC were observed for the CVRT group (ΔVAS, 6.72 ± 3.77; ΔABC, 56.63 ± 40.55), followed by the GVRT and VORX groups. The greatest reduction in DHI-total was observed for the GVRT group (Δ 63.00 ± 26.06), but the intergroup differences were not statistically significant (*p* = 1.000) (Table 3). Effect size analysis (η^2^) indicated small to moderate effects (η^2^ = 0.10–0.24) for between-group comparisons of primary outcomes, consistent with limited statistical power due to the small sample. These findings should therefore be interpreted as exploratory and hypothesis-generating rather than confirmatory. Overall, all three rehabilitation programs were associated with within-group improvements in dizziness and balance measures, with no statistically significant superiority of any single approach. No adverse events were observed during the study period. All interventions were well tolerated, and no participant discontinued due to adverse effects.

**Table 3:** Comparison of vestibular and balance function before and after treatment among three groups.

ΔScore	Group CVRT (*n* = 11) [95% CI]	Group GVRT (*n* = 6) [95% CI]	Group VORX (*n* = 6) [95% CI]	*p*-value
mCTSIB firm EO	0.45 ± 0.93 [−0.10, 1.00]	0	0	0.167
mCTSIB firm EC	1.00 ± 1.14 [0.33, 1.67]	0.00 ± 0.63 [−0.50, 0.50]	0.16 ± 0.75 [−0.44, 0.76]	0.251
mCTSIB foam EO	1.00 ± 1.26 [0.26, 1.74]	0.50 ± 0.83 [−0.16, 1.16]	1.00 ± 0.63 [0.50, 1.50]	0.656
mCTSIB foam EC	1.45 ± 1.12 [0.79, 2.11]	0.83 ± 0.41 [ 0.50, 1.16]	1.33 ± 1.21 [0.36, 2.30]	0.491
FRT	1.33 ± 9.62 [−4.36, 7.02]	4.66 ± 8.52 [−2.16, 11.48]	3.05 ± 6.14 [−1.86, 7.96]	0.348
VAS	6.72 ± 3.77 [4.49, 8.95]	6.33 ± 4.08 [3.07, 9.59]	3.83 ± 3.86 [0.74, 6.92]	0.400
Disability scale	1.72 ± 1.73 [0.70, 2.74]	2.66 ± 1.36 [1.57, 3.75]	1.83 ± 1.32 [0.77, 2.89]	0.349
DHI-total	45.27 ± 41.01 [21.03, 69.51]	63.00 ± 26.06 [42.15, 83.85]	30.66 ± 40.58 [−1.81, 63.13]	1.000
DHI-functional	17.45 ± 16.92 [7.45, 27.45]	28.66 ± 10.32 [20.40, 36.92]	14.66 ± 18.53 [−0.17, 29.49]	0.295
DHI-emotional	14.36 ± 14.44 [5.83, 22.89]	19.00 ± 11.64 [9.69, 28.31]	9.66 ± 14.55 [−1.98, 21.30]	0.340
DHI-physical	14.36 ± 11.37 [7.64, 21.08]	15.33 ± 6.28 [10.30, 20.36]	6.33 ± 9.50 [−1.27, 13.93]	0.227
ABC	56.63 ± 40.55 [32.67, 80.59]	48.15 ± 19.51 [32.54, 63.76]	15.61 ± 17.49 [1.62, 29.60]	0.054

Group CVRT, Customized exercise; Group GVRT, Cawthorn-Cooksey exercise; Group VORX, VOR adaptation.

Kruskal Wallis test, Values are presented as mean ± standard deviation (SD) with 95% confidence intervals (CI). *p < 0.05: significant.

## Discussion

VRT is widely recognized as an effective intervention for improving balance and reducing dizziness in patients with aUPVD. It facilitates rapid recovery of symptoms and functional abilities in patients with aUPVD, and it is recommended as a core component of treatment in clinical practice guidelines in several countries. However, only a few studies have directly compared different VRT approaches to determine the most effective method for patients with aUPVD despite its established role (12, 20).

This randomized controlled multicenter study investigated the effectiveness of three different VRT exercises in patients with aUPVD over an 8-week rehabilitation period: (1) a customized vestibular rehabilitation program (CVRT group) that included individualized combinations of gaze stabilization, postural balance, and gait training tailored to the symptoms and functional status of each patient; (2) a generic program using the standardized Cawthorne-Cooksey exercises (GVRT group); and (3) a program focused solely on VOR adaptation exercises (VORX group). All three interventions led to significant within-group improvements in dizziness severity, balance confidence, and functional disability, consistent with prior research supporting the general efficacy of VRT in promoting central vestibular compensation (31–33). Improvements in all groups likely reflect the natural process of vestibular compensation and the general benefits of structured rehabilitation, rather than the superiority of any single protocol. The present findings should be interpreted as preliminary, hypothesis-generating evidence due to the limited sample size and exploratory design, yet they provide useful comparative data to inform future large-scale trials. Following unilateral vestibular loss, the CNS adapts via mechanisms, such as adaptation, habituation, and substitution (1, 3). Vestibular rehabilitation plays a crucial role in accelerating this process by providing structured, movement-based interventions that promote sensory integration, balance control, and gaze stabilization.

Among the three groups, the CVRT group showed the most consistent and greatest improvements in both subjective and objective measures, particularly in VAS and ABC scores. Although these changes were not statistically significant, their magnitude suggests a potential benefit of individualized rehabilitation strategies. These findings align with previous studies suggesting that individualized rehabilitation approaches may enhance treatment outcomes by targeting patient-specific impairments (8, 17–19). The customized VRT protocol in this study represented a progression-based, symptom-guided framework that individualized exercise type and intensity according to each patient’s recovery status. Vestibular rehabilitation encompasses a broad range of multidisciplinary interventions, including medical management, patient education, and environmental adaptation. In contrast, vestibular physical therapy (VPT) refers specifically to exercise-based interventions aimed at promoting vestibular compensation through gaze stabilization, balance, and postural training (13). Because the present study focused exclusively on exercise-based programs, it can be more precisely described as a VPT intervention rather than a comprehensive VR program.

Customized vestibular rehabilitation enhances recovery by targeted interventions based on the specific impairments of an individual and providing tailored vestibular rehabilitation programs. Tailoring rehabilitation intensity and focus according to symptom patterns may improve functional recovery while maintaining patient engagement. For example, patients with severe postural instability may benefit more from balance-focused exercises, whereas those experiencing oscillopsia may require a stronger emphasis on VOR adaptation training (34). This has been found to be more effective in improving postural stability, reducing falls, and enhancing the overall quality of life relative to generic rehabilitation approaches (35). This adaptability may explain the superior performance observed in the CVRT group relative to the other two groups in this study. Clinical guidelines also have highlighted the importance of tailoring VRT to individual impairments and functional limitations (9, 10).

Multiple studies reinforce the clinical benefit of supervised and customized approaches. In a randomized controlled study by Shiozaki et al., supervised VRT demonstrated significant benefits in patients with chronic peripheral vestibular disorders. This study found that structured, therapist-guided VRT significantly improved subjective dizziness and physical as well as social activities (16). In a randomized pilot study of older patients with central vestibular dysfunction, Marioni et al. demonstrated that supervised vestibular rehabilitation combined with home exercises was more effective than home exercises alone. The supervised group showed greater improvements in balance and larger reductions in dizziness-related disability, particularly in the functional and emotional domains of the DHI (15). Another randomized clinical trial by Smółka et al. compared a customized supervised outpatient vestibular rehabilitation program with a home-based exercise protocol using the Cawthorne-Cooksey method. The customized program led to significantly greater improvements in balance outcomes, including scores on the Berg Balance Scale and Dynamic Gait Index, as well as reductions in dizziness severity as measured by the DHI and VAS (18). A recent study by Kellerer et al. demonstrated that patients receiving personalized physiotherapist instructions experienced significantly greater improvement in Dizziness Handicap Inventory (DHI) scores than those who followed generic booklets (19).

The superior performance of the CVRT and GVRT group may be explained by its multimodal and adaptive structure, which combines gaze stabilization, postural control, and gait training based on each patient’s functional status. Nevertheless, because statistical significance was not achieved between groups, these results should be interpreted as supportive of the overall effectiveness of VRT rather than evidence of a single superior method. Given this moderate statistical power (≈0.63), the findings should be regarded as preliminary trends rather than definitive evidence of superiority among rehabilitation protocols. Such multimodal programs are grounded in the principles of vestibular compensation—namely, adaptation (improving VOR function), substitution (utilizing alternative sensory inputs or postural strategy), and habituation (desensitizing to provocative movements (1, 3, 4). By addressing multiple components of vestibular dysfunction simultaneously, customized rehabilitation may optimize sensory integration and accelerate recovery. Within this framework, VOR x1 training (head movement toward a stationary target) is intended to strengthen gaze stability through vestibulo-ocular adaptation, whereas VOR x2 (head and target moving in opposite directions) further stresses visuo-vestibular integration and sensory reweighting under dynamic conditions. These complementary targets help contextualize why VOR-focused elements may aid dynamic visual stability yet still need to be combined with balance and gait tasks to address postural control (36).

In contrast, the VORX group, which focused exclusively on gaze stabilization, showed only modest improvement. While VOR adaptation exercises are essential for enhancing dynamic visual stability (37), they may not fully address other domains such as postural control or gait function, which are critical in the early recovery phase of aUPVD. Similarly, although the GVRT group using the Cawthorne-Cooksey protocol showed significant within-group improvements, the fixed progression and lack of personalization may limit its adaptability to diverse patient profiles (38, 39). The clinical presentation of the participants was consistent with acute vestibular neuritis, and the nystagmus pattern suggested mainly superior division involvement. However, because detailed vestibular testing was unavailable, the exact distribution of the lesion could not be verified. This limitation has been acknowledged, and future studies incorporating quantitative tests will allow more accurate differentiation of superior versus inferior division dysfunction. These findings support the concept that VRT effectiveness depends not only on the exercise type but also on the individualization, supervision, and intensity of practice (40).

The results of this study support the clinical utility of customized vestibular rehabilitation programs in patients with acute unilateral vestibulopathy (41). While generic rehabilitation programs and VOR adaptation exercises remain effective, customized VRT may have superior outcomes by tailoring exercises to individual needs. Given the exploratory nature of this trial, future multicenter studies with larger samples, prospective registration, and objective adherence monitoring are warranted to confirm these trends and to define optimal exercise dosing and timing. This has important implications for clinical practice, as vestibular rehabilitation programs should be designed to maximize patient-specific recovery, rather than rely solely on standardized exercise regimens (34, 42, 43).

In addition, the clinical presentation of the participants was consistent with acute vestibular neuritis, most likely involving the superior division, as suggested by the pattern of spontaneous horizontal–torsional nystagmus. However, because advanced vestibular function tests such as vHIT and VEMP were not performed uniformly across centers, the exact division of lesion involvement (superior vs. inferior) could not be confirmed. Future studies incorporating quantitative vestibular testing will be necessary to clarify the precise lesion site and its relationship with rehabilitation outcomes.

This study has several limitations. First, the number of participants was relatively small, and no *a priori* power calculation was performed, which may limit the generalizability and statistical robustness of the results. The trial was conducted as a multicenter pilot study, and several participant dropouts may have further reduced the statistical power to detect intergroup differences. The study was performed under institutional review board approval but was not prospectively registered, as the trial began before public registration was routinely implemented for investigator-initiated rehabilitation studies. Second, objective vestibular assessments such as vHIT, VEMP, or computerized dynamic posturography were not included, which may have limited the physiological interpretation of the outcomes. In addition, home exercise adherence was based on self-report rather than objective monitoring, and variations in exercise intensity or frequency may have influenced the results. Third, the follow-up period was limited to 8 weeks, so long-term effects and sustainability of the observed improvements remain uncertain. Finally, all participants were recruited from three centers within a single country, which may restrict the external validity of the findings. Future studies with larger, prospectively powered samples, improved participant retention, longer follow-up, and objective monitoring of vestibular and training outcomes are warranted to validate and extend these preliminary results.

## Conclusion

This multicenter randomized study compared three vestibular rehabilitation approaches for acute unilateral peripheral vestibular dysfunction. All interventions led to significant improvements in balance and dizziness symptoms during the 8-week rehabilitation period. The customized program showed a trend toward greater improvement, although differences among groups were not statistically significant. Early initiation of vestibular rehabilitation appears beneficial, and larger, prospectively registered studies with objective assessments are needed to confirm these preliminary findings.

## Data Availability

The raw data supporting the findings of this study are available from the corresponding author upon reasonable request.

## References

[1] Han BI Song HS Kim JS. Vestibular rehabilitation therapy: review of indications, mechanisms, and key exercises. J Clin Neurol. (2011) 7:184–96. doi: 10.3988/jcn.2011.7.4.184, 22259614 PMC3259492

[2] Huang HH Chen CC Lee HH Chen HC Lee TY Tam KW . Efficacy of vestibular rehabilitation in vestibular neuritis: a systematic review and meta-analysis. Am J Phys Med Rehabil. (2024) 103:38–46. doi: 10.1097/phm.0000000000002301, 37339059

[3] Lacour M Helmchen C Vidal PP. Vestibular compensation: the neuro-otologist's best friend. J Neurol. (2016) 263:S54–64. doi: 10.1007/s00415-015-7903-4 27083885 PMC4833803

[4] Tjernström F Zur O Jahn K. Current concepts and future approaches to vestibular rehabilitation. J Neurol. (2016) 263:65–70. doi: 10.1007/s00415-015-7914-1, 27083886 PMC4833789

[5] Lacour M Bernard-Demanze L. Interaction between vestibular compensation mechanisms and vestibular rehabilitation therapy: 10 recommendations for optimal functional recovery. Front Neurol. (2014) 5:285. doi: 10.3389/fneur.2014.00285, 25610424 PMC4285093

[6] Lacour M Tighilet B. Plastic events in the vestibular nuclei during vestibular compensation: the brain orchestration of a "deafferentation" code. Restor Neurol Neurosci. (2010) 28:19–35. doi: 10.3233/rnn-2010-0509, 20086280

[7] McDonnell MN Hillier SL. Vestibular rehabilitation for unilateral peripheral vestibular dysfunction. Cochrane Database Syst Rev. (2015) 1:Cd005397. doi: 10.1002/14651858.CD005397.pub4, 25581507 PMC11259236

[8] Aratani MC Ricci NA Caovilla HH Ganança FF. Benefits of vestibular rehabilitation on patient-reported outcomes in older adults with vestibular disorders: a randomized clinical trial. Braz J Phys Ther. (2020) 24:550–9. doi: 10.1016/j.bjpt.2019.12.003, 31952916 PMC7779949

[9] Rossi-Izquierdo M Gayoso-Diz P Santos-Pérez S Del-Río-Valeiras M Faraldo-García A Vaamonde-Sánchez-Andrade I . Short-term effectiveness of vestibular rehabilitation in elderly patients with postural instability: a randomized clinical trial. Eur Arch Otorrinolaringol. (2017) 274:2395–403. doi: 10.1007/s00405-017-4472-4, 28251319

[10] Tekin Dal B Bumin G Aksoy S Günaydın R. Comparison of activity-based home program and Cawthorne-Cooksey exercises in patients with chronic unilateral peripheral vestibular disorders. Arch Phys Med Rehabil. (2021) 102:1300–7. doi: 10.1016/j.apmr.2020.12.022, 33529612

[11] Hall CD Herdman SJ Whitney SL Cass SP Clendaniel RA Fife TD . Vestibular rehabilitation for peripheral vestibular hypofunction: an evidence-based clinical practice guideline: FROM THE AMERICAN PHYSICAL THERAPY ASSOCIATION NEUROLOGY SECTION. J Neurol Phys Ther. (2016) 40:124–55. doi: 10.1097/npt.0000000000000120, 26913496 PMC4795094

[12] Hall CD Herdman SJ Whitney SL Anson ER Carender WJ Hoppes CW . Vestibular rehabilitation for peripheral vestibular hypofunction: an updated clinical practice guideline from the Academy of neurologic physical therapy of the American Physical Therapy Association. J Neurol Phys Ther. (2022) 46:118–77. doi: 10.1097/npt.0000000000000382, 34864777 PMC8920012

[13] Whitney SL Sparto PJ. Principles of vestibular physical therapy rehabilitation. NeuroRehabilitation. (2011) 29:157–66. doi: 10.3233/nre-2011-0690, 22027077 PMC4894843

[14] Kim MK Yun SY Lee S Lee JO Sung SY Lee JY . Efficacy of vestibular rehabilitation and its facilitating and hindering factors from real-world clinical data. Front Neurol. (2024) 15:1329418. doi: 10.3389/fneur.2024.1329418, 38487329 PMC10938910

[15] Vereeck L Wuyts FL Truijen S De Valck C Van de Heyning PH. The effect of early customized vestibular rehabilitation on balance after acoustic neuroma resection. Clin Rehabil. (2008) 22:698–713. doi: 10.1177/0269215508089066 18678570

[16] Shiozaki T Ito T Wada Y Yamanaka T Kitahara T. Effects of vestibular rehabilitation on physical activity and subjective dizziness in patients with chronic peripheral vestibular disorders: a six-month randomized trial. Front Neurol. (2021) 12:656157. doi: 10.3389/fneur.2021.656157, 33995253 PMC8117149

[17] Marioni G Fermo S Lionello M Fasanaro E Giacomelli L Zanon S . Vestibular rehabilitation in elderly patients with central vestibular dysfunction: a prospective, randomized pilot study. Age (Dordr). (2013) 35:2315–27. doi: 10.1007/s11357-012-9494-7, 23179254 PMC3825000

[18] Smółka W Smółka K Markowski J Pilch J Piotrowska-Seweryn A Zwierzchowska A. The efficacy of vestibular rehabilitation in patients with chronic unilateral vestibular dysfunction. Int J Occup Med Environ Health. (2020) 33:273–82. doi: 10.13075/ijomeh.1896.01330, 32235946

[19] Kellerer S Amberger T Schlick C Dlugaiczyk J Wuehr M Jahn K. Specific and individualized instructions improve the efficacy of booklet-based vestibular rehabilitation at home - a randomized controlled trial (RCT). J Vestib Res. (2023) 33:349–61. doi: 10.3233/ves-220122, 37182850

[20] Kammerlind AS Ledin TE Odkvist LM Skargren EI. Effects of home training and additional physical therapy on recovery after acute unilateral vestibular loss--a randomized study. Clin Rehabil. (2005) 19:54–62. doi: 10.1191/0269215505cr830oa, 15704509

[21] Vander Vegt CB Hill-Pearson CA Hershaw JN Loftin MC Bobula SA Souvignier AR. A comparison of generalized and individualized vestibular rehabilitation therapy in a military TBI sample. J Head Trauma Rehabil. (2022) 37:380–9. doi: 10.1097/htr.0000000000000777, 35452022

[22] Allum JH Ledin T. Recovery of vestibulo-ocular reflex-function in subjects with an acute unilateral peripheral vestibular deficit. J Vestib Res. (1999) 9:135–44. doi: 10.3233/VES-1999-9208, 10378185

[23] Allum JH Honegger F. Recovery times of stance and gait balance control after an acute unilateral peripheral vestibular deficit. J Vestib Res. (2016) 25:219–31. doi: 10.3233/ves-150561, 26890423

[24] Teggi R Caldirola D Fabiano B Recanati P Bussi M. Rehabilitation after acute vestibular disorders. J Laryngol Otol. (2009) 123:397–402. doi: 10.1017/s0022215108002983, 18549515

[25] Tokle G Mørkved S Bråthen G Goplen FK Salvesen Ø Arnesen H . Efficacy of vestibular rehabilitation following acute vestibular neuritis: a randomized controlled trial. Otol Neurotol. (2020) 41:78–85. doi: 10.1097/mao.0000000000002443, 31789800

[26] Schubert MC. Vestibular, Rehabilitation In: Encyclopedia of computational neuroscience. New York, NY: Springer (2022). 3531–5.

[27] Shepard NT Telian SA Smith-Wheelock M. Habituation and balance retraining therapy. A retrospective review. Neurol Clin. (1990) 8:459–75. 2359384

[28] Jacobson GP Newman CW. The development of the dizziness handicap inventory. Arch Otolaryngol Head Neck Surg. (1990) 116:424–7. doi: 10.1001/archotol.1990.01870040046011, 2317323

[29] Park JW Shin YG Gu JW Song MH Shim DB. Compensation of the postural instability in patients with acute unilateral vestibular neuritis: the usefulness of computerized dynamic posturography as an objective indicator. Korean J Otorhinolaryngol-Head Neck Surg. (2017) 60:295–300. doi: 10.3342/kjorl-hns.2017.00374

[30] Wellons RD Duhe SE MacDowell SG Hodge A Oxborough S Levitzky EE. Estimating the minimal clinically important difference for balance and gait outcome measures in individuals with vestibular disorders. J Vestib Res. (2022) 32:223–33. doi: 10.3233/ves-201630, 35147571

[31] Horak FB Jones-Rycewicz C Black FO Shumway-Cook A. Effects of vestibular rehabilitation on dizziness and imbalance. Otolaryngol Head Neck Surg. (1992) 106:175–80. 1738550

[32] Sedeño-Vidal A Hita-Contreras F Montilla-Ibáñez MA. The effects of vestibular rehabilitation and manual therapy on patients with unilateral vestibular dysfunction: a randomized and controlled clinical study. Int J Environ Res Public Health. (2022) 19:19. doi: 10.3390/ijerph192215080, 36429797 PMC9690966

[33] Dunlap PM Holmberg JM Whitney SL. Vestibular rehabilitation: advances in peripheral and central vestibular disorders. Curr Opin Neurol. (2019) 32:137–44. doi: 10.1097/wco.0000000000000632, 30461465

[34] Maslovara S Butkovic-Soldo S Peric M Pajic Matic I Sestak A. Effect of vestibular rehabilitation on recovery rate and functioning improvement in patients with chronic unilateral vestibular hypofunction and bilateral vestibular hypofunction. NeuroRehabilitation. (2019) 44:95–102. doi: 10.3233/nre-182524, 30776020

[35] Whitney SL Alghadir AH Anwer S. Recent evidence about the effectiveness of vestibular rehabilitation. Curr Treat Options Neurol. (2016) 18:13. doi: 10.1007/s11940-016-0395-4, 26920418

[36] Ferri N Casagrande Conti L Manzari L Piatti D De Angelis D Nocentini U . Assessment of vestibulo-ocular reflex function in people with Parkinson's disease: a cross-sectional study in a rehabilitation setting using the video head impulse test. BMJ Open. (2025) 15:e099765. doi: 10.1136/bmjopen-2025-099765, 40764084 PMC12336473

[37] Roller RA Hall CD. A speed-based approach to vestibular rehabilitation for peripheral vestibular hypofunction: a retrospective chart review. J Vestib Res. (2018) 28:349–57. doi: 10.3233/ves-180633, 29689764 PMC9249287

[38] Szturm T Ireland DJ Lessing-Turner M. Comparison of different exercise programs in the rehabilitation of patients with chronic peripheral vestibular dysfunction. J Vestib Res. (1994) 4:461–79. doi: 10.3233/VES-1994-4606, 7850042

[39] Alahmari KA Sparto PJ Marchetti GF Redfern MS Furman JM Whitney SL. Comparison of virtual reality based therapy with customized vestibular physical therapy for the treatment of vestibular disorders. IEEE Trans Neural Syst Rehabil Eng. (2014) 22:389–99. doi: 10.1109/tnsre.2013.2294904, 24608691 PMC5527704

[40] Alashram AR. Effects of Cawthorne-Cooksey exercises on vestibular symptoms: a systematic review of randomized controlled trials. J Bodyw Mov Ther. (2024) 39:132–41. doi: 10.1016/j.jbmt.2024.02.026 38876618

[41] Hillier S McDonnell M. Is vestibular rehabilitation effective in improving dizziness and function after unilateral peripheral vestibular hypofunction? An abridged version of a Cochrane review. Eur J Phys Rehabil Med. (2016) 52:541–56. 27406654

[42] Tramontano M Princi AA De Angelis S Indovina I Manzari L. Vestibular rehabilitation in patients with persistent postural-perceptual dizziness: a scoping review. Hear Balance Commun. (2021) 19:282–90. doi: 10.1080/21695717.2021.1975986

[43] Castiglia SF Trabassi D Conte C Gioiosa V Sebastianelli G Abagnale C . Local dynamic stability of trunk during gait is responsive to rehabilitation in subjects with primary degenerative cerebellar Ataxia. Cerebellum. (2024) 23:1478–89. doi: 10.1007/s12311-024-01663-4, 38279000 PMC11269439

[44] Strupp M Arbusow V Maag KP Gall C Brandt T. Exercise treatment for vestibular compensation: A review. Journal of Neurology. (19987) 245:435–43. doi: 10.1080/21695717.2021.1975986

